# Assessment of the efficacy of the far cortical locking technique in proximal humeral fractures: a comparison with the conventional bi-cortical locking technique

**DOI:** 10.1186/s12891-020-03821-1

**Published:** 2020-12-02

**Authors:** Joong-Bae Seo, Jae-Sung Yoo, Yeon-Jun Kim, Kyu-Beom Kim

**Affiliations:** 1 Department of Orthopaedic Surgery, Dankook University College of Medicine, Cheonan, South Korea; 2 Department of Orthopaedic Surgery, Asan Chungmu Hospital, Mojongdong 432-2, Asan, Chungnam Republic of Korea

**Keywords:** Far cortical locking screw, Locking plate fixation, Proximal humeral fracture

## Abstract

**Background:**

Locking plate fixation is one of the treatment strategies for the management of proximal humeral fractures. However, stiffness after locking plate fixation is a clinical concern. The mechanical stiffness of the standard locking plate system may suppress the interfragmentary motion necessary to promote secondary bone healing by callus formation. The far cortical locking (FCL) technique was developed to address this limitation in 2005. FCL increases construct flexibility and promotes callus formation. Our study aimed to evaluate the clinical and radiological outcomes of the FCL technique when implemented in proximal humeral fracture management. Furthermore, we compared the surgical outcomes of FCL with those of the conventional bicortical locking (BCL) screw fixation technique.

**Methods:**

Forty-five consecutive patients who had undergone locking fixation for proximal humeral fractures were included in this study. A proximal humeral locking plate (PHILOS) system with BCL screw fixation was used in the first 27 cases, and the periarticular proximal humeral locking plate with FCL screw fixation was used in the final 18 consecutive cases. Functional capacity was assessed using the constant score, American Shoulder and Elbow Surgeons (ASES) score, and range of motion. Radiographic outcomes were evaluated using the Paavolainen method of measuring the neck-shaft angle (NSA).

**Results:**

No significant differences in clinical outcomes (ASES score, constant score, and range of motion) were found between the two groups. The union rate at 12 weeks was significantly higher in the FCL group (94.4%) than in the BCL group (66.7%, *p* = 0.006). No significant differences in NSA were found between the two treatment strategies. The complication rate was not significantly different between the two groups.

**Conclusions:**

When implemented in proximal humeral fractures, the FCL technique showed satisfactory clinical and radiological outcomes as compared with the conventional BCL technique. The bone union rate at 12 weeks after surgery was significantly higher in the FCL group than in the BCL group. However, no significant difference in the final bone union rate was found between the two groups.

## Background

Identification and implementation of the most efficacious treatments for proximal humeral fractures remain challenging [1, 2]. Although various techniques have been described, consensus has not been reached yet on the procedures offering the best clinical outcomes [1, 2]. Since the development of the locking plate and screw system, osteosynthesis has accounted for a greater percentage of treatments in patients with displaced proximal humeral fractures [3, 4]. However, while locking plates provide an adequate biological environment for healing, the locking plate have a tendency to be overly rigid and suppress callus formation, as shown by Bottlang et al. at the distal femur [5]. Several studies have suggested that locking plates interfere with callus formation, resulting in insufficient fracture healing, particularly in the near cortex [6, 7]. Furthermore, animal models and clinical studies alike have consistently shown that locked plating fixation leads to scarce and asymmetric callus formation around the near cortex [8, 9] owing to the lack of interfragmentary motion due to construct rigidity.

To address this problem, the far cortical locking (FCL) concept was developed. In FCL, the screws lock rigidly with the plate and far cortex while still allowing a controlled motion envelope on the near cortex [10]. FCL screws have a flexible shaft with a reduced diameter that can elastically deflect within the near cortex motion envelope, and the motion envelope is controlled by the diameter of a collar segment adjacent to the FCL screw head [11]. Recently, several studies have reported satisfactory clinical and radiological outcomes of this technique in periarticular knee fractures [12, 13].

.To the best of our knowledge, no in vivo studies have compared clinical and radiological outcomes between FCL and conventional bicortical locking (BCL) screw fixation in proximal humeral fractures. Therefore, the aim of this study was to analyze and compare the clinical and radiological outcomes of the FCL and BCL procedures in proximal humeral fractures. We hypothesized that the clinical outcomes would not be altered between the procedures, but the time to union in the FCL technique would be shorter than that in the BCL technique.

## Methods

This study was performed with approval from Dankook university hospital institutional research ethics committee and informed consent from all the patients (DKUH-2020-04-035). The procedures in this study were performed under the Declaration of Helsinki’s ethical principles for medical research involving human participants. Written informed consent was obtained from all individual participants included in the study. We enrolled 45 consecutive patients who had undergone an open-reduction and internal fixation procedure for a proximal humeral fracture at our institution during the period of January 2013 and May 2018.

Patients with 2-, 3-, or 4-part fractures were included in this study on the basis of the Neer classification [14]. Patients with surgical neck fractures deemed to be unstable by a surgeon were included. Exclusion from the study required that patients meet at least one of the following criteria: treatment with the minimally invasive plate osteosynthesis technique or fibular allograft, combined large to massive rotator cuff tears, pathological fractures, an irreparable head and/or tuberosity fragments, stable fractures with intact medial support, and an immature skeleton or loss to follow-up before 12 months of enrollment. The BCL screw fixation technique was used in the first 27 cases, and the FCL screw fixation technique was used in the final 18 consecutive cases. Patient information was obtained from the patients’ medical records, including age, sex, dominant hand, height, weight, body mass index, American Society of Anesthesiologists (ASA) classification, mechanism of injury, time to surgery, and smoking history.

### Description of the BCL screw fixation procedure

**Fig. 1:**
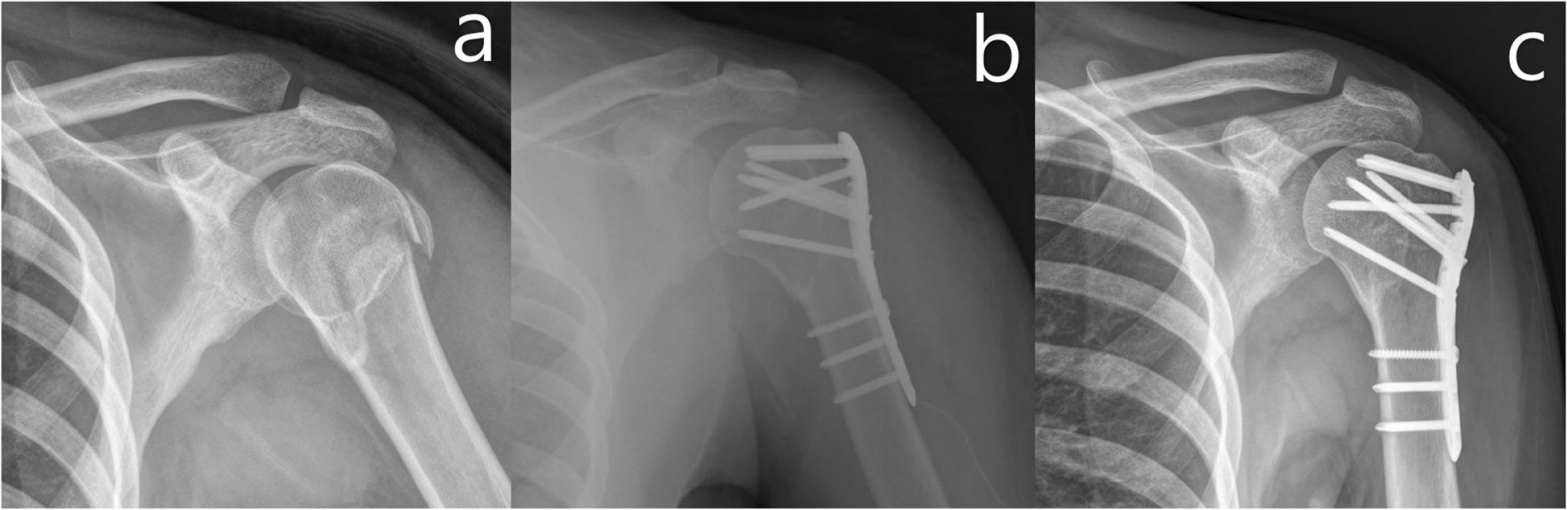
Simple radiographs of the conventional bicortical locking screw fixation. **a** Preoperative, **b** Postoperative and **c** 3 months after surgery

Under general anesthesia, all the patients underwent surgery in the supine position using the standard deltopectoral approach. The fracture fragments are first temporarily reduced with Kirschner wires, and then sutures are passed through the rotator cuff tendon and fixed with a proximal humeral internal locking system (PHILOS) plate (DePuy Synthes, Zuchwil, Switzerland) with one 3.5-mm cortical screw and two or three BCL screws (Fig. 1). After the PHILOS plate fixation, the 3.5-mm cortical screw is loosened from the longitudinal combi-hole of the plate shaft. The rotator cuff tendons are then attached with four to five No. 5 non-absorbable braided sutures (Ethibond, Somerville, NJ, USA). The sutures are then passed through the 3.5-mm cortical screw and tied off. Finally, the cortical screw is fastened fully to complete the procedure.

### Description of the FCL screw fixation procedure

**Fig. 2:**
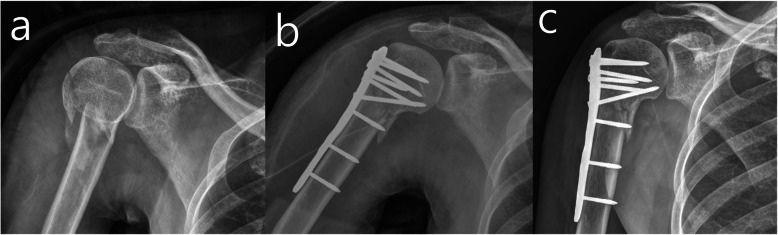
Simple radiographs of the far cortical locking system fixation. **a** Preoperative, **b** Postoperative and **c** 3 months after surgery

The same position, approach, reduction, and fixation are used in the FCL and BCL screw fixation procedures. After placement, the periarticular proximal humeral locking plate (Zimmer Biomet, Warsaw, Indiana, USA) is secured with three FCL screws, and the rotator cuff tendon is augmented with multiple sutures tied to the plate through the suture holes (Fig. 2).

### Postoperative rehabilitation and implant removal

After surgery, the affected arm was first kept in a splint for 1 week and then in a sling for the following 6 weeks. Pendulum, self-assisted circumduction, and gradual passive range of motion (ROM) exercises were implemented 1 week postsurgery if tolerated by the patient. Furthermore, 6 weeks postsurgery, active ROM exercises were implemented. Implant removal was conducted 3 months postsurgery, provided that bone union was accomplished. If the bone union was not complete at 3 months, implant removal was delayed until full union was observed. If the patient presented with stiffness of the shoulder joint, brisement was performed concomitantly at the time of implant removal.

### Clinical and radiological evaluations

Clinical assessments included the constant score, the American Shoulder and Elbow Surgeons (ASES) score, and range of motion. These assessments were recorded by the physician’s assistant at the final follow-up. Full range of motion was measured and documented, including active forward flexion, abduction, external rotation, and internal rotation at the back. An independent examiner blinded to all patient data evaluated the values at each postoperative follow-up visit.

**Fig. 3:**
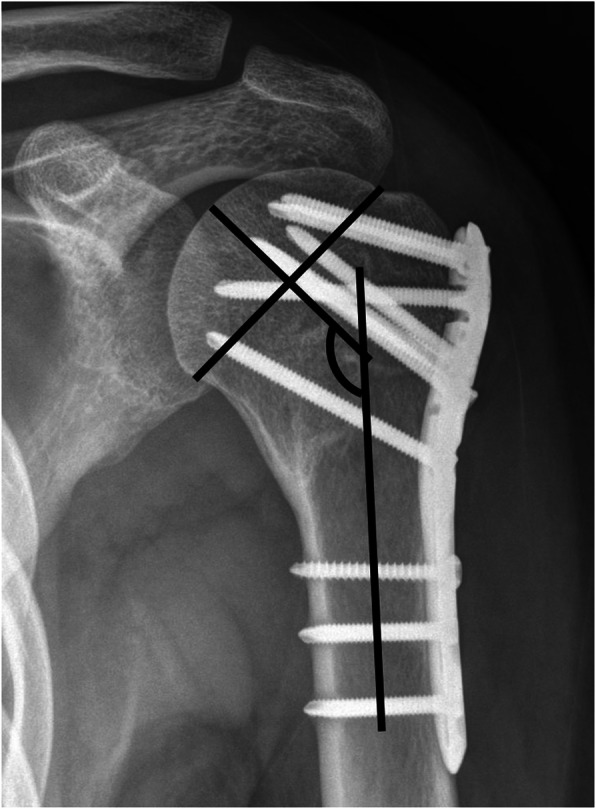
The neck-shaft angle was measured by drawing a line from the superior to the inferior border of the articular surface and then a perpendicular line through the center of the humeral head. The angle between this line and the line bisecting the humeral shaft was measured as the neck-shaft angle

The radiological evaluation included both clavicle and anteroposterior views taken at regular intervals after surgery (3, 6, and 12 weeks; 6 months; and 1 year post surgery). The radiographic outcomes included union rate, union at 12 weeks, and structural alignment. Bony union was defined by the existence of a bridging callus in not less than three cortices in two planes. Delayed union was defined as an incomplete radiographical consolidation after ≥6 months. Alignment was evaluated immediately after the procedure and 1 year after surgery, using the Paavolainen method [14], which measures the humeral neck-shaft angle (NSA). The NSA was determined as a line perpendicular to another line, between the superior and inferior borders of the articular surface on anteroposterior radiography. NSA is formed by the intersection of this perpendicular line and another line, bisecting the humeral shaft (Fig. 3) [14]. The NSA was analyzed by two independent examiners (J.K.K. and G.Y.K.). Individual values were measured, and the mean values were calculated. All complications were recorded, including fracture collapse, screw penetration, avascular necrosis of the humeral head, peri-implant fracture, postoperative infection, stiffness of the shoulder joint, and the necessity for additional surgery.

### Statistical analyses

To establish if the continuous data were normally distributed, the Kolmogorov-Smirnov test was used. An independent *t* test was performed to analyze continuous variables; and a Pearson chi-square test, to analyze non-continuous variables. All statistical analyses were performed through the Statistical Package for Social Sciences version 25.0 (SPSS, Inc., an IBM Co., IL, USA). A *p* value of < 0.05 denoted statistically significant differences in all the analyses.

## Results

Twenty-five women and 20 men were included in this study, with an age range of 18–84 years. The initial injuries were caused by a traffic accident in 12 cases, falls in 27, and sports-related injuries in 6. No significant differences were found between the two surgical groups in terms of demographic data and time to surgery, except for the mean follow-up period (Table 1).

**Table 1:** Demographic data

Variable	Bicortical locking group (*n* = 27)	Far cortical locking group (*n* = 18)	*p*-value
Mean age	53.0 ± 12.4	56.7 ± 15.8	0.759
Gender (Male: Female)	12: 15	8: 10	0.335
Dominant arm: Non-dominant arm	14: 13	8: 10	0.626
Height (cm)	162.1 ± 7.7	161.4 ± 7.8	0.791
Weight (kg)	64.8 ± 11.2	64.6 ± 12.3	0.951
Body mass index	24.7 ± 3.8	24.7 ± 3.8	0.965
Smoking: Non-smoking	6: 21	2: 16	0.340
ASA class (1:2:3)	9: 14: 4	4: 10: 4	0.663
Mechanism of Injury			0.885
Traffic accident	8	4	
Fall	15	12	
Sport injury	4	2	
Neer classification (2:3:4)	5: 14: 8	6: 8: 4	0.535
Time to surgery (day)	4.0 ± 2.6	3.7 ± 2.3	0.626
Mean follow-up (month)	15.9 ± 6.0	12.7 ± 1.9	0.031

*ASA* American Society of Anesthesiologists

No significant differences in ASES score, constant score, and range of motion were found between the two groups. The mean ASES scores were 79.2 ± 18.6 and 80.4 ± 14.8 in the BCL and FCL groups, respectively. The mean constant scores were 73.3 ± 15.1 and 75.2 ± 12.2 in the two groups, respectively. The mean forward elevation was 122.8 ± 17.7 and 128.3 ± 16.8; mean external rotation, 30.0 ± 11.9 and 30.6 ± 11.7; and the mean internal rotation, T10 and T11 in the BCL and FCL groups, respectively (Table 2).

**Table 2:** Clinical and Radiologic outcomes between the two groups

Variable	Bicortical locking group (*n* = 27)	Far cortical locking group (*n* = 18)	*p*-value
Union at 12 weeks (%)	18 (66.7%)	17 (94.4%)	0.006
Neck-shaft angle			
Postoperative	135.9 ± 6.1	137.6 ± 12.9	0.267
1 year later surgery	133.2 ± 6.7	136.4 ± 11.9	0.257
Paavolainen Classification			0.521
Good (130° ± 10°)	24	17	
Fair (100° - 120°)	3	1	
Poor (< 100°)	0	0	
ASES score (100)	79.2 ± 18.6	80.4 ± 14.8	0.845
Constant score (100)	73.3 ± 15.1	75.2 ± 12.2	0.629
Range of motion			
Forward elevation	122.8 ± 17.7	128.3 ± 16.8	0.296
External rotation	30.0 ± 11.9	30.6 ± 11.7	0.878
Internal rotation	T10	T11	0.576

*ASES* American Shoulder and Elbow Surgeons

Union was observed 12 weeks after surgery in 18 (66.7%) of the 27 patients in the BCL group. The remaining 8 patients (29.6%) showed bony union 6 months after surgery and 1 patient (3.7%) showed bony union at 1 year post-surgery. Healing took longer during the follow-up period in the BCL group than in the FCL group. Union was observed 12 weeks after surgery in 17 (94.4%) of the 18 patients in the FCL group. One patient (5.6%) showed bony union at 6 months postsurgery. One case of delayed union was observed in the BCL group, and no metal failure or nonunion occurred in either group (Table 2).

The mean NSA in the BCL group was 135.9° ± 6.1° postoperatively and 133.2° ± 6.7° at 1-year follow-up. The mean NSA of the FCL group was 137.6° ± 12.9° postoperatively and 136.4° ± 11.9°at 1 year follow-up. These values were not statistically significantly different between the BCL and FCL groups (*p* = 0.267 and *p* = 0.257, respectively). The Paavolainen method revealed that 41 cases (91.1%) presented with a good mean NSA value of 130° ±10°, while 4 (8.9%) presented with a fair mean NSA value of 100–120. However, no significant difference in mean NSA value was found between the two groups (*p* = 0.521; Table 2).

Fourteen patients (31.1%) had complications, including shoulder stiffness (*n* = 10), avascular necrosis of humeral head with screw penetration (*n* = 1), and migration of the greater tuberosity (*n* = 3). A single patient presented with shoulder stiffness 1 year after the initial surgery and underwent brisement concomitantly with plate removal. This resulted in a satisfactory outcome during the final follow-up. No further complications occurred, such as wound aggravation, or infection (Table 3).

**Table 3:** Complications between the two groups

Variable	Bicortical locking group (*n* = 27)	Far cortical locking group (*n* = 18)
Overall complications (n, %)	9 (33.3%)	5 (27.8%)
Fracture collapse (%)	–	–
Metal failure (%)	–	–
Screw loosening (%)	–	–
Infection (%)	–	–
Peri-hardware fracture (%)	–	–
Stiffness at 1 year after surgery (%)	6 (22.2%)	4 (22.2%)
Avascular necrosis with screw penetration (%)	1 (3.7%)	–
Resorption or migration of the greater tuberosity (%)	2 (7.4%)	1 (5.6%)

## Discussion

The primary aim of this study was to assess the clinical and radiological outcomes of treating proximal humeral fractures with open reduction and internal fixation strategies. The present comparison between the conventional BCL and FCL techniques in proximal humeral fractures showed that the patients treated with the FCL technique had satisfactory clinical and radiological outcomes, but no significant differences in the clinical outcomes or complications between the two groups. Supporting our initial hypothesis, the bone union rate at 12 weeks postsurgery was significantly higher in the FCL group than in the BCL group. However, no significant difference in the final bony union rate was observed between the two groups.

Proximal humeral fracture is the third most common osteoporotic fragility fracture type in elderly patients and can be a critical factor in overall morbidity and functional loss [15, 16]. From 1999 to 2005, the incidence of proximal humeral fractures was roughly 250 per 100,000 Medicare patients [16]. Eighty percent of the patients with proximal humeral fractures were female aged between 80 and 89 years. Furthermore, most operative cases fell within the 74- to 84-year age range [15, 16]. Furthermore, osteoporotic bone significantly increases the risk of proximal humeral fractures, with 2.6 times greater risk of fracture in osteoporosis patients (12.1 per 1000 woman-years) than in non-osteoporosis patients (4.6 per 1000 woman-years) [17]. Achieving and maintaining a reduction and adequate hardware fixation are often challenging in these patients because of the thin cortical bone, crushed cancellous bone, and comminution due to poor bone quality [18].

The locking plate fixation method has consistently improved biomechanical efficacy as compared with the other methods of fixation in osteoporotic bone [19, 20]. However, the stiffness caused by the locking plate was a concern, and patients might not have obtained the required interfragmentary motion sufficient to induce callus formation for fracture healing [7, 11]. Therefore, surgery-related complications such as fixation loss, implant failure, infection, and both delayed union and nonunion may occur due to the plate stiffness [21]. In 2005, the FCL technique was developed to address plate stiffness and the associated complications. When compared with the conventional locking plating techniques, FCL has been demonstrated to enable a more flexible fixation [22]. The increased flexibility of the FCL structures facilitates secondary bone healing and improved fracture union [22]. The FCL system differs from the conventional locked screws; at the near cortex, the core diameter of the MotionLoc screw (Zimmer, Warsaw, IN) is smaller and does not engage the plate closest to the cortex, thereby providing the necessary motion at the near cortex [5, 8, 10, 22]. The clinical impact of the FCL technique on long fractures was confirmed by several biomechanical studies that showed multiple benefits of reducing axial stiffness by providing approximately parallel interfragmentary micro motion [5, 8, 10, 22].

Clinical studies have provided strong evidence to support the advantages of the FCL technique in fracture management, indicating multiple benefits such as safety and effectiveness [12, 13, 23–25]. In 2014, Bottlang et al. [23] demonstrated that FCL was both safe and effective in treating a series of 31 distal femoral fractures. In their study, the primary endpoint was the union of the fracture without any complications or secondary intervention; 30 of the 31 cases showed favorable outcomes without any treatment revision [23]. In 2015, Adams et al. [12] performed a retrospective analysis in 15 patients with fractures at the distal femur who underwent surgery using MotionLOC screws. The mean time of bone union in the entire group was 24 weeks, and the authors provided compelling data that demonstrated that the FCL technique was effective for fractures of the distal femur, with high nonunion rates, as compared with the traditional locking constructs [13]. In 2017, Madey et al. [24] prospectively evaluated 11 patients who had humeral shaft fractures and underwent surgery with an active locking plate without any additional bone graft or bone morphogenic proteins. Ten of the 11 cases showed a healing period of 10.9 ± 5.2 weeks, as evident by the bridging of the callus and restored physiological functioning without pain. The authors also suggested that the dynamic fixation accompanied by the active locking plates may accelerate fracture healing than the standard locking plate techniques. They concluded that this was likely due to early callus bridging and accelerated recovery of function [24]. In 2019, Wang et al. [25] retrospectively evaluated 144 patients with lower limb fractures; 76 patients were treated with the FCL technique and 68 patients were treated with a standard plating technique. The researchers found that while the FCL system resulted in improved early callus formation, no evidence showed that the FCL system had better outcomes than the standard plating techniques in terms of final fracture healing, complications associated with surgery, or functional outcomes [25].

While several clinical studies have examined FCL screw fixation, to the best of our knowledge, the present study may be the first in vivo assessment that compared clinical and radiological outcomes between of the FCL and BCL fixation techniques in proximal humeral fracture management. In the present study, the time for bone union was significantly shorter in the FCL group than in the conventional BCL group. However, no significant differences in the radiologic assessment of alignment, clinical outcomes, and surgical complications were found between the two groups.

The present study has several limitations. First, this study was conducted as a non-randomized retrospective study. Second, only 18 patients were included in the FCL group. A small sample size often leads to type II error due to the low statistical power, although a retrospective power analysis concluded that a minimum of 16 cases were required in each group to identify a 10% difference between the groups, with an *α* level of 0.05 and *β*value of 0.80.Third, patients with polytrauma, including those with ipsilateral upper extremity fractures, were not excluded. Therefore, the comparison of clinical outcomes between the two groups was limited. Fourth, rotator cuff suture fixation methods and plate design were not the same between two groups, this can be a cause of bias in this study. Fifth, patients with 2-, 3-, or 4-part fractures were included. It also can be a cause of bias in this study, although only unstable surgical neck fractures were included and there was no difference of Neer classification between two groups. Sixth, CT scan is the gold standard for evaluating bone union. However, union was defined using only simple radiography without CT evaluation. To increase the accuracy of the union assessment, cases in which the 2 shoulder fellows did not agree were not defined as union. Seventh, there is a possibility of decreasing the FCL system effect in FCL fixation group, because calcar screw crossed fracture line in some cases. Finally, although previous studies showed that osteoporosis is a contributing factor to surgical outcomes, the effect of osteoporosis could not be evaluated in the present study because a bone matrix density analysis could not be performed in all the cases.

## Conclusion

When conducted in proximal humeral fractures, the FCL screw fixation technique showed satisfactory clinical and radiological outcomes than the established BCL screw fixation technique. In addition, the bone union rate at 12 weeks postsurgery was significantly higher in the FCL group than in the BCL group. However, no significant difference in final bone union rate was found between the two groups.

## Data Availability

The datasets generated during this current study are available from the corresponding author upon reasonable request.

## Abbreviations

FCL: Far cortical locking
BCL: Bicortical locking
PHILOS: Proximal humeral locking plate
ASES: American Shoulder and Elbow Surgeons
NSA: Neck-shaft angle
ASA: American Society of Anesthesiologists
ROM: Range of motion
